# Proteomic analysis of halotolerant proteins under high and low salt stress in *Dunaliella salina* using two-dimensional differential in-gel electrophoresis

**DOI:** 10.1590/1678-4685-GMB-2015-0108

**Published:** 2016-05-13

**Authors:** Yan-Long Jia, Hui Chen, Chong Zhang, Li-Jie Gao, Xi-Cheng Wang, Le-Le Qiu, Jun-Fang Wu

**Affiliations:** 1 Pharmacy College, Xinxiang Medical University, Xinxiang 453003, Henan, China; 2 Henan Collaborative Innovation Center of Molecular Diagnosis and Laboratory Medicine, Xinxiang Medical University, Xinxiang 453003, Henan, China; 3 School of Basic Medicine, Xinxiang Medical University, Xinxiang 453003, Henan, China

**Keywords:** Dunaliella salina, halotolerant protein, proteomics, two-dimensional differential in-gel electrophoresis

## Abstract

*Dunaliella salina*, a single-celled marine alga with extreme salt tolerance, is an important model organism for studying fundamental extremophile survival mechanisms and their potential practical applications. In this study, two-dimensional differential in-gel electrophoresis (2D-DIGE) was used to investigate the expression of halotolerant proteins under high (3 M NaCl) and low (0.75 M NaCl) salt concentrations. Matrix-assisted laser desorption ionization time-of-flight mass spectrometry (MALDI-TOF/TOF MS) and bioinformatics were used to identify and characterize the differences among proteins. 2D-DIGE analysis revealed 141 protein spots that were significantly differentially expressed between the two salinities. Twenty-four differentially expressed protein spots were successfully identified by MALDI-TOF/TOF MS, including proteins in the following important categories: molecular chaperones, proteins involved in photosynthesis, proteins involved in respiration and proteins involved in amino acid synthesis. Expression levels of these proteins changed in response to the stress conditions, which suggests that they may be involved in the maintenance of intracellular osmotic pressure, cellular stress responses, physiological changes in metabolism, continuation of photosynthetic activity and other aspects of salt stress. The findings of this study enhance our understanding of the function and mechanisms of various proteins in salt stress.

## Introduction

Salt stress is a major natural abiotic stress and plants have evolved sophisticated mechanisms to adapt to saline environments (Zhang *et al*., 2012). *Dunaliella salina*, a unicellular eukaryotic alga, can survive in environments containing 0.5-5 M NaCl (Mishra *et al*., 2008). Various studies have used salt-tolerant algae as model organisms to investigate the mechanisms of salt tolerance (Liska *et al*., 2004; Oren, 2014). In addition to the study of glycerin synthesis in several salt-tolerant algal species (Goyal, 2007), some proteins involved in adaptation to salt have been isolated, *e.g*., membrane structures associated with heat shock proteins 70 and 90 (HSP70 and HSP90, respectively), glucose-6-phosphate dehydrogenase and nitrate reductase (Liska *et al*., 2004; Katz *et al*., 2007; Lao *et al*., 2014). However, there is little genetic bioinformation about these proteins, which limits further research. High-throughput proteomics is a powerful tool for in-depth exploration of the mechanism of salt tolerance in algae (Liska *et al*., 2004). Gel electrophoresis, particularly two-dimensional differential in-gel electrophoresis (2D-DIGE) (Tonge *et al*., 2001), has been used to simultaneously analyze multiple samples that are imaged separately in order to detect protein differences of < 10% at the 95% confidence level. When combined with mass spectrometry (Katz *et al*., 2007), this technique can precisely determine molecular mass and analyze the molecular structure (Hu *et al*., 2005; Jin *et al*., 2007; Brechlin *et al*., 2008).

In the present study, proteomic analysis by 2D-DIGE was used to investigate the total protein content of *D. salina* cultured under two levels of salinity. A differential protein expression map, mass spectrometry and bioinformatics analysis were used to analyze and identify the differentially expressed proteins in order to improve our understanding of their function in salt tolerance.

## Materials and methods

### Algal culture

*Dunaliella salina* (UTEX-LB-1644, Culture Collection of Algae, University of Texas, USA) was cultured in modified medium at low (0.75 M NaCl) or high (3 M NaCl) salinity at 26 °C and an illumination of 4500 Lux for 12 h/day (Ben-Amotz and Avron, 1990). Before being used, cultured algae were examined microscopically to ensure that the cells were axenic, motile and flagellated, and that cell debris was minimal. Viability curves for *D. salina* cells in different NaCl concentrations over time were obtained to ensure that a high salt stress (3 M NaCl) did not affect cell growth. *Dunaliella salina* cells in the logarithmic phase of growth (density: ~2 × 10^6^ cells/mL) were collected for further analysis.

### Protein extraction, desalting, freeze-drying and quantification

*Dunaliella salina* proteins were isolated using the methods of Hirano *et al*. (2006) and Natarajan *et al*. (2005), with minor modifications (Jia *et al*., 2010). Initially, 2 mL of ice-cold freezing solution (10 mM Tris-MPOS, 2 mM MgCl_2_ and 10 mM KCl at pH 7.5) was added to a tube containing *D. salina* (2 × 10^8^ cells), mixed and the suspension placed in liquid nitrogen for 2 min. Three freeze-thaw cycles were applied to thoroughly lyse the cells. Next, 6 mL of ice-cold TCA/acetone buffer [acetone with 10% (w/v) TCA and 0.07% (w/v) β-mercaptoethanol] was added, the proteins were precipitated at −20 °C overnight, and the tubes were then centrifuged (20,000 *g*, 15 min, 4 °C). The supernatant was decanted, the pellet was washed with chilled wash buffer [acetone with 0.07% (w/v) β-mercaptoethanol and 2 mM EDTA] plus 0.5 mL of a protease inhibitor cocktail (Sigma) to a final volume of 50 mL and the acetone mixture was then removed by centrifugation. The pellet was re-suspended in buffer [7 M urea, 2 M thiourea, 4% (w/v) CHAPS, 0.5% Bio-Lyte 3/10 Ampholyte (BIO-RAD) and 65 mM dithiothreitol (DTT)] and 1% (v/v) P9599 protease inhibitor cocktail (Sigma) to a final volume of 50 mL, incubated at 4 °C for 30 min with occasional vortex mixing, and then centrifuged (20,000 *g*, 20 min, 10 °C). The total protein extracted from *D. salina* by this procedure was either immediately subjected to further analysis or stored in aliquots at −80 °C.

The extracted proteins from *D. salina* were desalted, freeze-dried and concentrated. A disposable PD-10 desalting column (GE Healthcare, Munich, Germany) was used to recover a desalted sample according to the manufacturers instructions. Since the total volume of each sample increased to about 3.5 mL during desalting, the samples were again concentrated by freeze-drying. The protein lysate was reconstituted and stored at -20 °C. The protein concentration was determined by the Bradford protein assay, using bovine serum albumin (BSA) as the standard.

### Protein labeling and 2D-DIGE analysis

The *D. salina* protein samples were labeled with fluorescent CyDyes for DIGE (Cy2, Cy3 and Cy5; GE Healthcare), according to the manufacturer's instructions. The order of staining with the dyes was altered among the protein samples in order to avoid artefacts caused by preferential labelling. Briefly, 50 μg of protein sample (pH 8.5) was labelled with 400 pmol of Cy3 or Cy5 minimal dye according to the experimental design, while a pool consisting of the same amount of each sample was labeled with Cy2 as an internal standard to control for quantitative comparisons. All of the individual samples were biological replicates. Protein sample labeling was done on ice in the dark for 30 min and then quenched by incubation with 1 μL of 10 mM L-lysine (GE Amersham Biosciences) on ice in the dark for 10 min. The labeled samples were then analyzed by 2D-DIGE.

Electrophoresis was done as described by Alban *et al*. (2003) and Tonge *et al*. (2001). Reagents and equipment used for DIGE were purchased from GE Healthcare. For each gel in DIGE, the protein samples labeled with Cy2, Cy3 or Cy5 (50 μg each) were pooled and an equal volume of rehydration buffer (8 M urea, 4% CHAPS, 2% DTT and 2% IPG buffer pH3-10) was added (the final concentration of DTT and IPG buffer was 1%). Isoelectric focusing of the pooled protein samples was done on non-linear IPG strips (24 cm long, pH 3-10) using an Ettan II IPG-phor apparatus (GE Healthcare). The strips were rehydrated at 30 V for 12 h at room temperature and isoelectric focusing was done at 500 V for 0.5 h, followed by 1000 V for 0.5 h, 4000 V for 2 h, 10000 V for 3 h and then 10000 V 70 h to reach a total of 70 Kvh. After isoelectric focusing, the strips were incubated for 15 min in equilibration buffer [50 mM Tris-HCl, 6 M urea, 20% (v/v) glycerol and 2% (w/v) SDS supplemented with 1% (w/v) dithiothreitol] and then for 15 min in 2.5% (w/v) iodoacetamide. The proteins were separated on 12.5% SDS-PAGE gels at 10 mA/gel for 15 min and then at 20 mA/gel at 20 °C until they reached the end of the plate. The analysis of cell lysates was done using at least three independent replicates and the protein spots used for comparisons were detected on all of the gels.

### Scanning of electrophoretic patterns and image analysis

The maps labeled with Cy2, Cy3 or Cy5 fluorescent dye were scanned with a Typhoon 9410 scanner (GE Healthcare) at wavelengths of 488/520 nm, 532/580 nm and 633/670 nm, respectively. Scan values ranged from 60,000 to 90,000 units, with differences of ~5,000 units among the three replicate gels for each sample. DeCyder v.5.02 DIGE image analysis software was used to analyze the images (DIA and BVA) and to identify the spots that differed between the high and low salinity treatments. When the presence of protein spots differed between the salinity treatments in at least two of the three analyzed gels (*i.e*., in six of nine analyzed images), this was designated a significant change. In addition, when the ratio between the standardized average spot volumes exceeded 1.5, this was statistically significant using Student's *t*-test at p < 0.05.

### Protein identification by mass spectrometry (MS)

The differentially expressed protein spots of interest were further identified by MS. Briefly, unlabeled pooled protein samples (800 μg) of each salinity group were run in parallel on separate preparative polyacrylamide gels and stained with Coomassie brilliant blue (Colloidal Blue stain kit; Invitrogen, Carlsbad, CA, USA) to facilitate MS analysis. The spots of interest were selected and manually cut out from the preparative gels. Tryptic digests were prepared according to the manufacturer's instruction. Briefly, the excised gel pieces containing the proteins of interest were destained by ultrasound with 25 mM NH_4_HCO_3_ (Fluka, USA) in 50% acetonitrile (ACN) (Merck, Germany) for 10 min and then lyophilized. Fifteen microliters of digestion buffer [10 ng of trypsin/μL (Promega, Madison, WI, USA) in 25 mM NH_4_HCO_3_] was added and the samples were digested overnight at room temperature. Peptides were extracted twice with 5% trifluoroacetic acid (TFA; ACROS, Belgium) for 1 h each and with 2.5% TFA/50% acetonitrile (ACN) for 1 h. The extracted peptides were pooled, dried completely by centrifugal lyophilization and re-suspended in 0.1% TFA. Equal volumes of the sample solution and CHCC matrix (5 mg/mL, dissolved in 50% ACN/0.1% TFA; Sigma-Aldrich, USA) were mixed and spotted onto the matrix-assisted laser desorption/ionization (MALDI) target plate.

Samples were analyzed using MALDI-time-of-flight (TOF)/TOF MS with a proteomics analyzer (4800 plus, Applied Biosystems SCIEX, USA). Mono-isotopic peak masses were acquired in a mass range of 700 to 4,000 Da. Ten of the most intense ion signals (signal/noise ratio or S/N > 20), excluding common trypsin autolysis peaks and matrix ion signals, were selected as precursors for MS/MS acquisition. Protein identification of the peptide mass fingerprint combined MS/MS data was done using Global Proteome Server (GPS) Explorer software (version 3.6, Applied Biosystems SCIEX, Framingham, MA, USA) with the NCBI non-redundant protein database (ncbi2009). The search parameters were set as follows: Taxonomy - all or plant, Enzyme - trypsin, peptide mass tolerance - ± 100 ppm, Fragment ion mass tolerance - ± 0.2 Da, Max missed cleavages - 2, Static modification - Carbamidomethyl (C) (57.021 Da), Dynamic modification - M oxidation (15.995 Da). The criterion for successful identification of proteins was a 95% confidence interval (95%CI) for protein scores and peptide mass fingerprint and MS/MS data. The results were further confirmed in the SwissProt protein database (SwissP.sprot_1105).

### Validation of selected proteins by western blotting

To further validate the alterations of selected proteins identified in the proteomic analysis, we examined the expression of glutamine synthetase (GS) by western blotting of protein samples from low and high salt conditions. Briefly, after determining protein concentrations by the Bradford method, protein samples were boiled in loading buffer (60 mM Tris-HCl, pH 6.8, 25% glycerol, 2% SDS, 14.4 mM β-mercaptoethanol) for 5 min. Equal amounts of protein (20 μg/well) of each sample were then separated by electrophoresis in a 12% SDS-polyacrylamide gel and electrotransferred onto a polyvinylidene difluoride (PVDF) membrane (Millipore, Bedford, MA, USA). After blocking with 5% (w/v) non-fat milk in TBST (20 mM Tris-HCl, pH7.6, 136 mM NaCl and 0.1% Tween-20) for 1 h at room temperature and rinsing, the blot was incubated overnight at 4 °C with goat polyclonal anti-GS (1:500; sc-6640, Santa Cruz, CA, USA) as primary antibody. The membranes were then washed four times with TBST (5?min each) and incubated at room temperature for 1.5 h with anti-goat secondary horseradish peroxidase-conjugated antibody (1:2000, SC-2768, Santa Cruz, CA, USA). After incubation with BeyoECL Plus (Beyotime Biotechnology, Nantong, China), the bands were visualized by using a ChemiDoc-It^®^2 810 Imager (UVP) and quantified by densitometric analysis. As an internal control for protein loading, the blots were stripped and probed with a mouse monoclonal anti-β-actin antibody (1:2000; SC-2048, Santa Cruz); the resulting immunoreactive bands were used to normalize the densities of the GS bands.

### Statistical analysis

The results were expressed as the mean ± standard deviation (SD), where appropriate. Statistical comparisons of the protein levels between the two groups were done using Student's unpaired *t*-test and one-way analysis of variance (ANOVA), with a value of p < 0.05 indicating significance. All of these statistical analyses were done using SPSS 13.0 software (SPSS, Chicago, IL, USA).

## Results and Discussion

**Figure 1:**
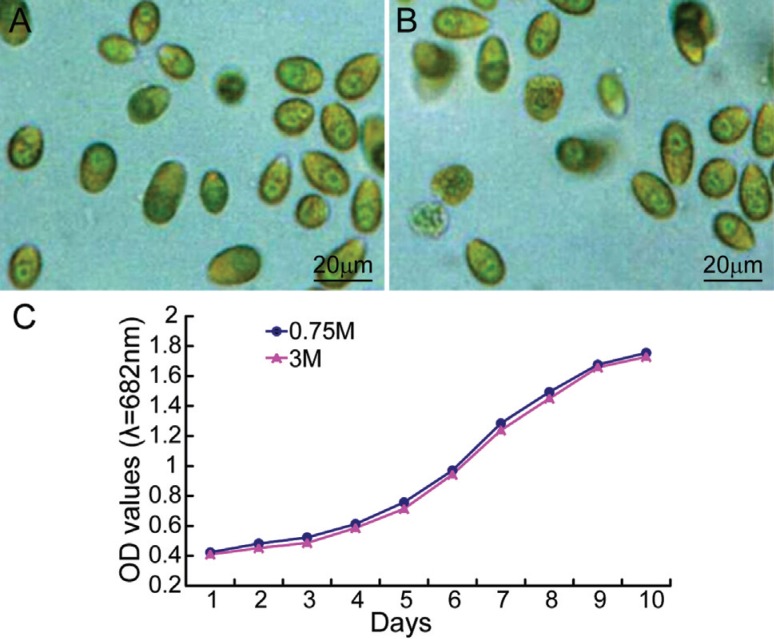
The appearance (A,B) and growth rate (C) of *D. salina* cells in high salinity (3 M) (A) and low salinity (0.75 M) (B) were very similar.

Sample preparation is a very important step in proteomics. We therefore initially examined the cell morphology of *D. salina* microscopically and determined the viability curves in different salt concentrations over time. Figure 1 shows that *D. salina* grew well and showed similar morphology and growth curves over time in both salinities. These findings indicated that *D. salina* cultured in low and high salinities was suitable for further study.

**Figure 2:**
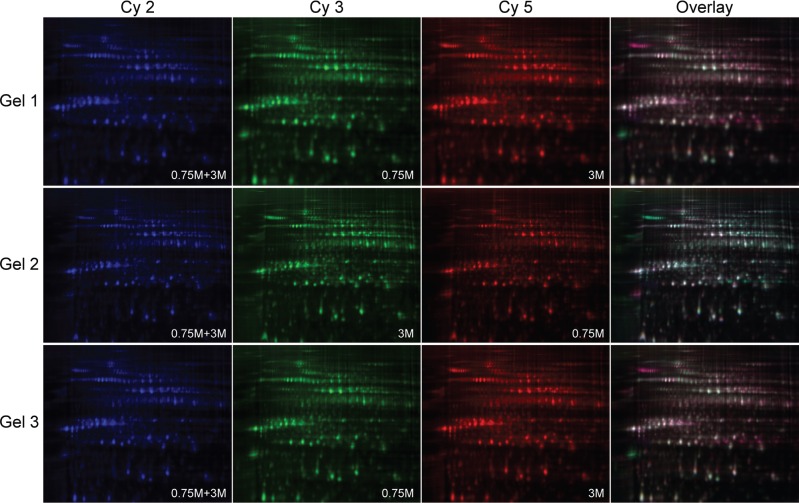
2-D DIGE gel images of proteins after exposure to salt stress in high (3 M) and low (0.75 M) salinity. The proteins were labeled with Cy2 [column 1, a pool (0.75M + 3M) consisting of the same amount of each sample as an internal standard to control for quantitative comparisons, scanning at wavelength of 488/520 nm], Cy3 (column 2, scanning at wavelength of 532/580 nm) or Cy5 (column 3, scanning at wavelength of 633/670 nm) fluorescent dyes; column 4 is an overlay of the first three columns. Gel1, 2, 3: triplicate gels for protein samples to reduce the gel-to-gel variations. Gel1: Cy2(0.75M + 3M) + Cy3(0.75M) + Cy5(3M); Gel2: Cy2(0.75M + 3M) + Cy3(3M) + Cy5(0.75M); Gel3, Cy2(0.75M + 3M) + Cy3(0.75M) + Cy5(3M).

**Figure 3:**
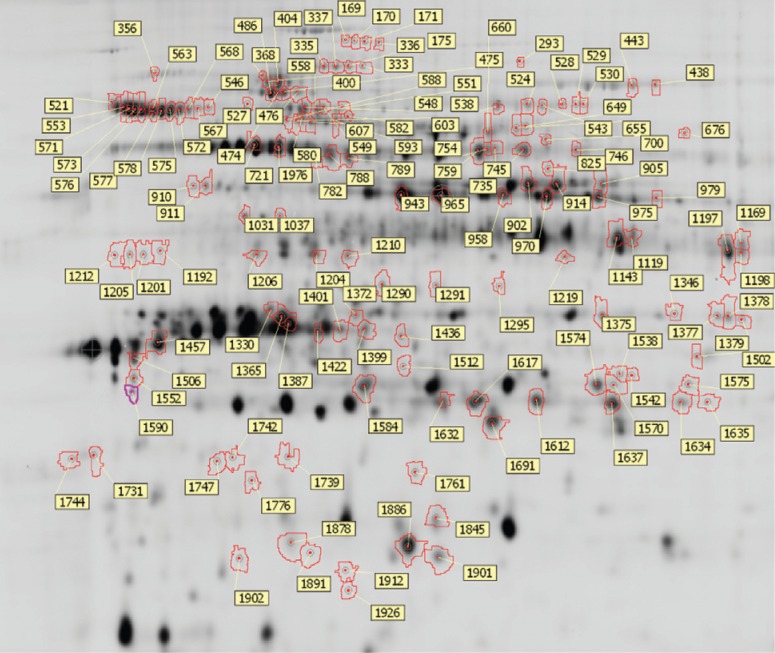
Differentially expressed *D. salina* protein spots identified by DIGE analysis between high and low salinity (3 M/0.75 M NaCl). Based on detailed image analysis, spot numbers with differences in standardized average spot volume ratios > 1.5 and a t-test < 0.05 are shown.

In order to extend our understanding of the molecular mechanisms of halotolerance in *D. salina*, we undertook a comparative proteomic analysis of *D. salina* grown in high (3 M) and low (0.75 M) salinity. As shown in Figure 2, the 2D gels of protein samples from *D. salina* grown in 3 M and 0.75 M NaCl were labeled with the fluorescent dyes Cy2, Cy3 or Cy5. Based on detailed image analysis, 141 protein spots that differed between the 3 M and 0.75 M salinity treatments were observed (Figure 3, Table 1). Further analysis of these 141 spots identified 33 spots containing 20 proteins that differed between the salinity treatments (Table 2). Among these proteins, heat shock protein (HSP), the α,β subunit of mitochondrial ATP synthase, GS, the light-harvesting protein of photosystem II, major light-harvesting complex II protein m7, sedoheptulose-1,7-bisphosphatase (SBPase), chlorophyll a-b binding protein of LHCII, and aspartate aminotransferase were up-regulated in high salinity (3 M), whereas α-tubulin, β-tubulin 2, major light-harvesting chlorophyll a/b protein 3 and ribulose-1,5-bisphosphate carboxylase/oxygenase (Rubisco) small subunit were down-regulated.

**Table 1:** Protein spots with significant changes between the treatment with 3 M and 0.75 M NaCl. The changes are expressed as the ratio 3 M/0.75 M.

Position	Master number	*t*-test	Average ratio (3 M/0.75 M)
1	575	1.80E-07	6.34
2	553	5.60E-07	11.06
3	1878	8.10E-07	−1.69
4	573	1.30E-06	9.87
5	1201	1.70E-06	5.77
6	1634	1.80E-06	5.26
7	576	2.20E-06	9.18
8	333	2.30E-06	1.97
9	529	2.40E-06	2.04
10	571	3.40E-06	9.13
11	475	5.60E-06	2.15
12	1205	6.30E-06	1.82
13	1635	7.00E-06	4.03
14	1617	7.20E-06	4.65
15	551	7.50E-06	1.92
16	567	7.80E-06	5.23
17	1744	7.80E-06	−5.72
18	593	9.70E-06	−1.8
19	746	1.20E-05	2.25
20	443	1.40E-05	1.55
21	1377	1.40E-05	1.59
22	1290	1.70E-05	1.73
23	1612	1.80E-05	6.8
24	336	2.00E-05	1.94
25	524	2.10E-05	2.17
26	546	2.40E-05	3.09
27	474	3.00E-05	1.9
28	577	3.40E-05	4.61
29	578	3.40E-05	4.78
30	905	3.40E-05	1.71
31	970	3.60E-05	2.12
32	486	3.80E-05	1.61
33	1169	3.80E-05	1.6
34	1378	3.90E-05	1.66
35	1575	4.40E-05	1.88
36	572	4.60E-05	6.21
37	1590	4.90E-05	1.92
38	975	5.10E-05	2.17
39	1584	5.50E-05	−1.59
40	1197	5.80E-05	1.66
41	754	6.20E-05	2.07
42	1206	6.50E-05	1.76
43	170	6.60E-05	1.86
44	538	7.20E-05	1.9
45	1731	7.50E-05	−5.31
46	438	7.70E-05	1.7
47	521	8.10E-05	7.1
48	943	8.70E-05	−1.78
49	528	9.70E-05	2.3
50	979	0.0001	1.65
51	1291	0.00011	1.84
52	527	0.00013	2.1
53	1346	0.00013	1.51
54	1542	0.00013	2.04
55	580	0.00014	−1.93
56	902	0.00014	1.56
57	1538	0.00014	1.86
58	1037	0.00016	2.02
59	1192	0.00016	1.58
60	1379	0.00016	1.74
61	958	0.00017	1.66
62	563	0.00018	5.63
63	1574	0.00018	1.73
64	582	0.00019	−1.63
65	649	0.00019	2.03
66	700	0.00021	−1.87
67	1143	0.00021	1.58
68	568	0.00022	6.68
69	789	0.00024	−1.53
70	1691	0.00025	−1.51
71	914	0.00026	1.68
72	175	0.00029	1.6
73	337	0.00029	2.05
74	549	0.00031	2.04
75	788	0.00031	−1.54
76	1632	0.00031	1.8
77	1637	0.00033	3.47
78	607	0.00034	−1.84
79	911	0.00035	−1.64
80	1119	0.00035	1.59
81	735	0.00036	1.53
82	1912	0.00038	2.63
83	476	0.00039	1.65
84	1901	0.00039	−2.36
85	368	0.00044	−1.88
86	1295	0.00044	1.62
87	1365	0.00044	1.69
88	588	0.00046	−1.77
89	676	0.00046	3.17
90	1502	0.00047	−1.58
91	1761	0.00047	2.47
92	1330	0.00049	1.53
93	782	0.0005	−1.72
94	530	0.00055	1.86
95	825	0.00055	1.56
96	1401	0.00055	1.97
97	400	0.00059	−2.13
98	1375	0.00059	−1.66
99	603	0.00061	−2.05
100	1512	0.00062	−1.57
101	759	0.00066	1.82
102	404	0.0007	−1.6
103	1552	0.00072	1.94
104	543	0.00074	1.73
105	1422	0.00076	1.51
106	1387	0.00078	1.52
107	1976	0.00079	−1.76
108	1031	0.001	2.03
109	745	0.0012	2.49
110	1204	0.0012	1.55
111	558	0.0013	1.52
112	1747	0.0013	−1.55
113	721	0.0014	1.57
114	1210	0.0014	1.73
115	1198	0.0016	1.78
116	1212	0.0017	1.99
117	335	0.0021	1.58
118	1570	0.0021	1.77
119	1776	0.0022	−2.26
120	548	0.0023	1.56
121	1399	0.0023	1.51
122	660	0.0028	1.5
123	1739	0.0029	−2.27
124	1926	0.0035	−2.25
125	1891	0.0038	−2.46
126	1886	0.0045	−1.62
127	171	0.0047	2
128	1372	0.0061	−1.71
129	1506	0.0064	1.63
130	910	0.0067	−1.59
131	1436	0.0067	−1.53
132	169	0.0082	1.82
133	1457	0.0089	−1.59
134	1902	0.0092	2.18
135	1219	0.01	1.66
136	655	0.012	1.52
137	293	0.013	1.65
138	356	0.013	−1.59
139	1845	0.013	1.9
140	965	0.017	−1.7
141	1742	0.022	−1.64

**Table 2:** Summary of 24 differentially expressed protein spots identified by MALDI-TOF/TOF MS after DIGE analysis.

Protein spot no.	Protein name	Accession no.	Protein score	Protein score (CI%)	Mr (Da)	Up/Down 3 M/0.75 M
548	ATP synthase subunit beta	gi|231586	288	100	60221.3	↑
549	Mitochondrial F-1-ATPase subunit 2 [*Zea mays*]	gi|162462751	284	100	59066.9	↑
551	Putative ATP synthase beta subunit [*Oryza sativa*]	gi|56784991	281	100	45879.8	↑
558	Beta subunit of mitochondrial ATP synthase	gi|159466892	271	100	61783	↑
721	ATP synthase beta-subunit [*Astrephomene*]	gi|4519320	375	100	26225.6	↑
582	Heat shock protein [*Dunaliella salina*]	gi|18250906	91	99.987	71708.9	↓
745	ATP synthase CF1 alpha subunit [*Chlamydomonas*]	gi|41179050	203	100	54717.7	↑
**??**	Adenosine triphosphatase [*Chlamydomonas reinhardtii*]	gi|1334356	193	100	48678.6	↑
746	ATP synthase CF1 alpha subunit [*Chlamydomonas*]	gi|41179050	151	100	54717.7	↑
**??**	Adenosine triphosphatase [*Chlamydomonas reinhardtii*]	gi|1334356	193	100	48678.6	↑
754	ATP synthase CF1 alpha subunit [*Chlamydomonas*]	gi|41179050	206	100	54717.7	↑
759	ATP synthase CF1 alpha subunit [*Chlamydomonas*]	gi|41179050	245	100	54717.7	↑
**??**	Adenosine triphosphatase [*Chlamydomonas reinhardtii*]	gi|1334356	213	100	48678.6	↑
782	Beta tubulin 2 [*Chlamydomonas reinhardtii*]	gi|159471706	299	100	49586.8	↓
1976	Beta tubulin 2 [*Chlamydomonas reinhardtii*]	gi|159471706	496	100	49586.8	↓
788	Alpha-tubulin [*Chloromonas sp*. ANT3]	gi|2625154	402	100	49536.6	↓
789	Alpha-tubulin [*Chloromonas sp*. ANT3]	gi|2625154	325	100	49536.6	↓
979	Glutamine synthetase [*Dunaliella tertiolecta*]	gi|3869304	89	99.979	22550.9	↑
1143	Aspartate aminotransferase Asp2 [*Arabidopsis thaliana*]	gi|22135928	67	96.233	22042.1	↑
1206	Sedoheptulose-1 7-bisphosphatase precursor [*Oryza*]	gi|27804768	104	100	42218.1	↑
1365	Chlorophyll a-b binding protein of LHCII	gi|115828	140	100	29089.4	↑
1387	Chlorophyll a-b binding protein of LHCII	gi|115828	237	100	29089.4	↑
1401	Light-harvesting protein of photosystem II	gi|159471686	107	100	26633.6	↑
1422	Light-harvesting protein of photosystem II	gi|159471686	128	100	26633.6	↑
1457	Major light-harvesting chlorophyll a/b protein 3	gi|123316054	165	100	27794.2	↓
1506	Major light-harvesting complex II protein m7	gi|19423289	96	99.996	27936.3	↑
1886	Ribulose-1,5-bisphosphate carboxylase/oxygenase small subunit	gi|44890111	73	98.986	21145.6	↓

Note: Protein scores with a CI% ≥ 95 were considered significant (p < 0.05) under the established criterion. Theoretical Mr (Da) are based on the amino acid sequences of the identified proteins.

Most plants can adapt to low or moderate salinity (Hasegawa *et al*., 2000). However, *D. salina* can adapt to a wide range of salt concentrations. In recent years, several studies have used proteomic or genomic methods to identify proteins of *D. salina* that are affected by salinity (Liska *et al*., 2004; Liu *et al*., 2014). Previous work concentrated on subcellular structures such as chloroplasts and the plasma membrane as demonstrated by Katz *et al*. (2007). Although knowledge of the genomics and protein sequences of *D. salina* is very limited, the analytical approach described here (*i.e*., extraction of total protein of *D. salina*, 2D-DIGE analysis, comprehensive analysis of differences in protein expression under high- and low-salt conditions and the identification of 20 proteins) may contribute to our understanding of the physiological processes of salt adaptation, as suggested by Pick (1992). Salt stress leads to multiple changes in basic biological functions such as photosynthesis, photorespiration and the synthesis of amino acids and carbohydrates (Kawasaki *et al*., 2001; Ozturk *et al*., 2002; Seki *et al*., 2002). The proteins identified in the present work also revealed that exposure to high salt in the environment altered the expression of many *D. salina* proteins involved in physiological and biochemical processes such as photosynthesis, stress defense, metabolism, molecular chaperones and cell structure. The functional significance and potential roles of the differentially expressed proteins associated with halotolerance in *D. salina* are discussed below.

HSPs are a family of proteins that are produced by cells in response to different environmental stress conditions, including exposure to heat shock, cold, UV light, nitrogen deficiency or water deprivation (Li and Srivastava, 2004). Therefore, up-regulation of HSP in *D. salina* can also be described more generally as part of the stress response (Santoro, 2000). In contrast, α-tubulin and β-tubulin 2 are down-regulated in *D. salina* under high salinity (3 M). We suspect that down-regulation of tubulin in response to high salinity may result in reduced cell motility, but further experiments are required to verify the hypothesis.

**Figure 4:**
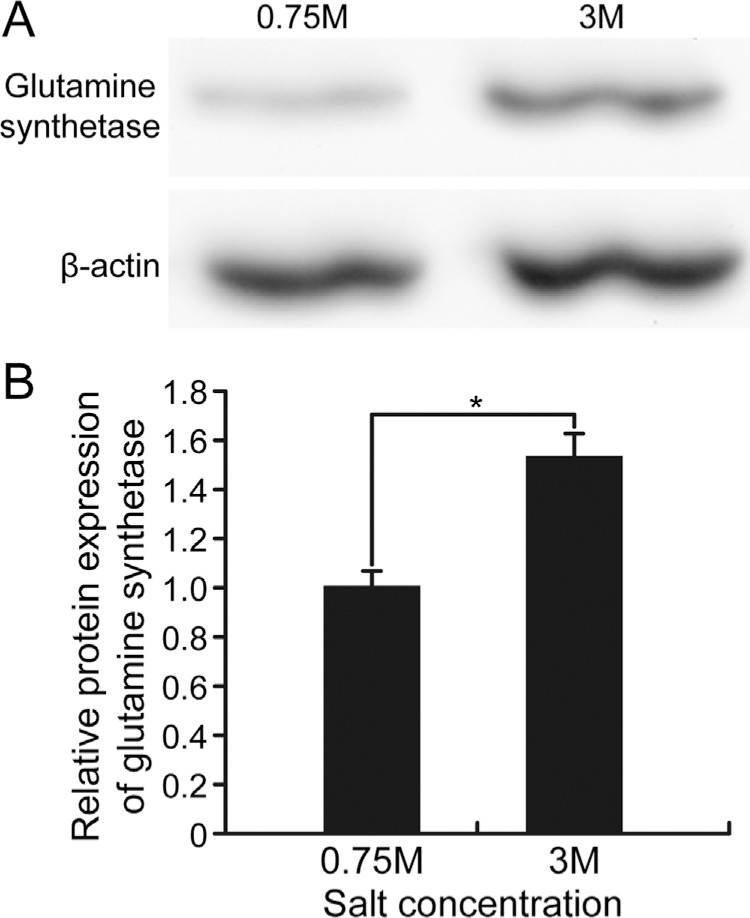
Protein expression of glutamine synthetase (GS) and β-actin in *D. salina* exposed to low (0.75 M NaCl) and high (3 M NaCl) salinity, as assessed by western blotting. (A) Representative bands of GS and β-actin in low (0.75 M) and high (3 M) salt conditions. Each lane contained 20 μg of protein. The experiments were run at least in triplicate and β-actin was used as an internal housekeeping gene. (B) Semi-quantitative densitometric analysis of immunoreactive bands of GS. Band intensity was normalized relative to β-actin. The columns represent the mean ± SD of three experiments. *p < 0.05.

As shown in Figure 4, western blotting, used to validate the enhanced expression of GS, confirmed that the expression of this protein was increased by exposure to high salinity in comparison to low salinity. This finding confirmed that obtained by MALDI-TOF/TOF MS after DIGE analysis. GS plays an essential role in nitrogen metabolism. Previous studies showed that overexpression of chloroplast GS could enhance tolerance to salt stress in transgenic rice (Hoshida *et al*., 2000) and may potentially be used to enhance the use of nitrogen, light and photorespiration in transgenic crop plants (Oliveira *et al*., 2002). The elevated expression of GS seen here in *D. salina* may play an important role in alleviating late-occurring salt stress and in maintaining the carbon-nitrogen metabolic balance during normal cell development and growth, as described by Bao *et al*. (2015).

Some of the proteins identified here were associated with photosynthesis and the Calvin cycle, including light harvesting protein of photosystem II, chlorophyll a-b binding protein, Rubisco and SBPase. Salt stress has been shown to inhibit photosynthesis in halophytes and non-halophytes, with the degree of inhibition being positively correlated with the salt concentration (Xu *et al*., 2000). For maximum efficiency, plants and green algae use chlorophyll a/b-binding proteins that can switch between being light-harvesting antenna for two photosystems (photosystem I and photosystem II) thereby providing an optimal balance in excitation (Kargul and Barber, 2008). Rubisco, a key enzyme involved in photosynthetic CO_2_ assimilation (Wang *et al*., 2015), is highly regulated in response to fluctuations in the environment, including changes in irradiance (Grabsztunowicz *et al*., 2015). SBPase is the most important factor for ribulose-1,5-bisphosphate (RuBP) regeneration in the Calvin cycle. An increase in the SBPase content of chloroplasts had a marked positive effect on photosynthesis (Tamoi *et al*., 2006). In the present study, the upregulated synthesis and activities of proteins related to photosynthesis and stress defense in *D. salina* may contribute to the priming effects that allow the cells to cope with salt stress. In addition to proteins related to photosynthesis, the up- or down-regulation of proteins involved in biochemical metabolism such as carbon and nitrogen metabolism in *D. salina* under salt stress, indicated that these processes were also differentially regulated.

In this work, we used a precipitation/resolubilization protocol for protein extraction. Theoretically, it is possible that the differences observed in the expression of certain proteins between low and high salt conditions could have reflected the inefficient resolubilization of some proteins, with the result that the insoluble residue was unintentionally eliminated. Close monitoring of resolubilization is therefore a critical step in sample preparation in order to ensure that all proteins are recovered. As shown elsewhere (Davidi *et al*., 2015), the insoluble pellet can be reextracted with 1% SDS, or other methods of purification that do not involve precipitation can be used for comparative proteomic analyses.

In summary, the level of many proteins in *D. salina* was altered in response to environmental salt stress. These proteins may be involved in maintaining intracellular osmotic pressure, cellular stress responses, physiological changes in metabolism, the continuation of photosynthesis, and other aspects of salt stress. These findings extend our understanding of the changes in protein expression associated with salt stress and provide new insights into the mechanisms of halotolerance in *D. salina*.

## References

[B1] Alban A, David SO, Bjorkesten L, Andersson C, Sloge E, Lewis S, Currie I (2003). A novel experimental design for comparative two dimensional gel
analysis: Two-dimensional difference gel electrophoresis incorporating a pooled
internal standard. Proteomics.

[B2] Bao A, Zhao Z, Ding G, Shi L, Xu F, Cai H (2015). The stable level of glutamine synthetase 2 plays an important role in
rice growth and in carbon-nitrogen metabolic balance. Int J Mol Sci.

[B3] Ben-Amotz A, Avron M (1990). The biotechnology of cultivating the halotolerant alga
*Dunaliella*. Trends Biotechnol.

[B4] Brechlin P, Jahn O, Steinacker P, Cepek L, Kratzin H, Lehnert S, Jesse S, Mollenhauer B, Kretzschmar HA, Wiltfang J (2008). Cerebrospinal fluid optimized two-dimensional difference gel
electrophoresis (2-D DIGE) facilitates the differential diagnosis of
Creutzfeldt-Jakob disease. Proteomics.

[B5] Davidi L, Levin Y, Ben-Dor S, Pick U (2015). Proteome analysis of cytoplasmatic and plastidic β-carotene lipid
droplets in *Dunaliella bardawil*. Plant Physiol.

[B6] Goyal A (2007). Osmoregulation in *Dunaliella*, part II: Photosynthesis
and starch contribute carbon for glycerol synthesis during a salt stress in
*Dunaliell atertiolecta*. Plant Physiol Biochem.

[B7] Grabsztunowicz M, Górski Z, Lucinski R, Jackowski G (2015). A reversible decrease in ribulose 1,5-bisphosphate
carboxylase/oxygenase carboxylation activity caused by the aggregation of the
enzyme's large subunit is triggered in response to the exposure of moderate
irradiance-grown plants to low irradiance. Physiol Plant.

[B8] Hasegawa PM, Bressan RA, Zhu JK, Bohnert HJ (2000). Plant cellular and molecular responses to high
salinity. Annu Rev Plant Physiol Plant Mol Biol.

[B9] Hirano M, Rakwal R, Shibato J, Agrawal GK, Jwa NS, Iwahashi H, Masuo Y (2006). New protein extraction/solubilization protocol for gel-based
proteomics of rat (female) whole brain and brain regions. Mol Cells.

[B10] Hoshida H, Tanaka Y, Hibino T, Hayashi Y, Tanaka A, Takabe T, Takabe T (2000). Enhanced tolerance to salt stress in transgenic rice that
overexpresses chloroplast glutamine synthetase. Plant Mol Biol.

[B11] Hu Y, Malone JP, Fagan AM, Townsend RR, Holtzman DM (2005). Comparative proteomic analysis of intra- and interindividual variation
in human cerebrospinal fluid. Mol Cell Proteomics.

[B12] Jia Y, Xue L, Li J, Liu H (2010). Isolation and proteomic analysis of the halotolerant alga
*Dunaliella salina flagella* using shotgun
strategy. Mol Biol Rep.

[B13] Jin T, Hu LS, Chang M, Wu J, Winblad B, Zhu J (2007). Proteomic identification of potential protein markers in cerebrospinal
fluid of GBS patients. Eur J Neurol.

[B14] Kargul J, Barber J (2008). Photosynthetic acclimation: Structural reorganisation of light
harvesting antenna - Role of redox-dependent phosphorylation of major and minor
chlorophyll a/b binding proteins. FEBS J.

[B15] Katz A, Waridel P, Shevchenko A, Pick U (2007). Salt-induced changes in the plasma membrane proteome of the
halotolerant alga *Dunaliella salina* as revealed by blue native
gel electrophoresis and nano-LC-MS/MS analysis. Mol Cell Proteomics.

[B16] Kawasaki S, Borchert C, Deyholos M, Wang H, Brazille S, Kawai K, Galbraith D, Bohnert HJ (2001). Gene expression profiles during the initial phase of salt stress in
rice. Plant Cell.

[B17] Lao YM, Jiang JG, Luo LX (2014). Characterization and expression patterns of nitrate reductase from
*Dunaliella bardawil* under osmotic stress and dilution
shock. Appl Biochem Biotechnol.

[B18] Li Z, Srivastava P (2004). Heat-shock proteins. Curr Protoc Immunol Appendix 1:Appendix 1T.

[B19] Liska AJ, Shevchenko A, Pick U, Kata A (2004). Enhanced photosynthesis and redox energy production contribute to
salinity tolerance in *Dunaliella* as revealed by homology-based
proteomics. Plant Physiol.

[B20] Liu J, Zhang D, Hong L (2014). Isolation, characterization and functional annotation of the salt
tolerance genes through screening the high-quality cDNA library of the halophytic
green alga *Dunaliella salina* (Chlorophyta). Ann Microbiol.

[B21] Mishra A, Mandoli A, Jha B (2008). Physiological characterization and stress-induced metabolic responses
of *Dunaliella salina* isolated from salt pan. J Ind Microbiol Biotechnol.

[B22] Natarajan S, Xu C, Caperna TJ, Garrett WM (2005). Comparison of protein solubilization methods suitable for proteomic
analysis of soybean seed proteins. Anal Biochem.

[B23] Oliveira IC, Brears T, Knight TJ, Clark A, Coruzzi GM (2002). Overexpression of cytosolic glutamine synthetase. Relation to
nitrogen, light, and photorespiration. Plant Physiol.

[B24] Oren A (2014). The ecology of *Dunaliella salina* in high-salt
environments. J Biol Res (Thessalon).

[B25] Ozturk ZN, Talame V, Deyholos M, Michalowski CB, Galbraith DW, Gozukirmizi N, Tuberosa R, Bohnert HJ (2002). Monitoring large-scale changes in transcript abundance in drought- and
salt-stressed barley. Plant Mol Biol.

[B26] Pick U, Avron M, Ben-Amotz A (1992). ATPases and ion transport in *Dunaliella*. *Dunaliella*: Physiology, Biochemistry and Biotechnology.

[B27] Santoro MG (2000). Heat shock factors and the control of the stress
response. Biochem Pharmacol.

[B28] Seki M, Ishida J, Narusaka M, Fujita M, Nanjo T, Umezawa T, Kamiya A, Nakajima M, Enju A, Sakurai T (2002). Monitoring the expression pattern of around 7,000
*Arabidopsis* genes under ABA treatments using a full-length
cDNA microarray. Funct Integr Genomics.

[B29] Tamoi M, Nagaoka M, Miyagawa Y, Shigeoka S (2006). Contribution of fructose-1, 6-bisphosphatase and
sedoheptulose-1,7-bisphosphatase to the photosynthetic rate and carbon flow in the
Calvin cycle in transgenic plants. Plant Cell Physiol.

[B30] Tonge R, Shaw J, Middleton B, Rowlinson R, Rayner S, Young J, Pognan F, Hawkins E, Currie I, Davison M (2001). Validation and development of fluorescence two-dimensional
differential gel electrophoresis proteomics technology. Proteomics.

[B31] Wang Y, Stessman DJ, Spalding MH (2015). The CO_2_ concentrating mechanism and photosynthetic carbon
assimilation in limiting CO_2_: How *Chlamydomonas* works
against the gradient. Plant J.

[B32] Xu XM, Ye HC, Li GF (2000). Progress in research of plant tolerance to saline
stress. Chinese J Appl Environ Biol.

[B33] Zhang HB, Wang T, Chen S, Li H, Zhang Y, Dai S (2012). Mechanisms of plant salt response: Insights from
proteomics. J Proteome Res.

